# *In situ* aluminium ions regulation for quantum efficiency and light stability promotion in white light emitting material†

**DOI:** 10.1039/c9ra01763a

**Published:** 2019-05-17

**Authors:** Qian Liu, Qi-Long Wu, Man-Xiu Nie, Da-Shuai Zhang, Jiong-Peng Zhao, Fu-Chen Liu

**Affiliations:** School of Chemistry and Chemical Engineering, Tianjin Key Laboratory of Organic Solar Cells and Photochemical Conversion, Tianjin University of Technology Tianjin 300384 China zhaojp@tjut.edu.cn fuchenliutj@yahoo.com; College of Chemistry and Chemical Engineering, Dezhou University Dezhou 253023 P. R. China

## Abstract

In this study, we have proposed an *in situ* ion regulation strategy to assemble a white-light-emitting material with high stability and efficiency. A fluorescence tunable hybrid material was first fabricated by a “ship around the bottle” method in which the fluorescent dyes, disodium 2-naphthol-3,6-disulfonate (R) and ZnO Quantum Dots (QDs), were embedded into metal–organic frameworks (MOFs) in proportion. Then, the competition coordination of aluminium ions over zinc ions to R were utilized to subtly adjust the intensity of blue fluorescence, leading to an ideal white light with Commission Internationale de l'Eclairage (CIE) coordinates of (0.30, 0.33) and a high Color-Rendering Index (CRI) value of 93%. Compared with the material fabricated by the ratio tuning of the R salt and ZnO QDs directly, the *in situ* ions regulation strategy enabled the final product to have a higher quantum efficiency and light stability. Moreover, this strategy also settled the non-tunable problem of fluorescence due to the competition coordination effects of aluminium ions and zinc ions in the same synthetic system. This synthetic strategy and our new findings can provide more ideas for designing new white-light-emitting materials.

## Abstract 2

An *in situ* ion regulation strategy to assemble white-light-emitting material with high stability and efficiency.

## Graphical abstract

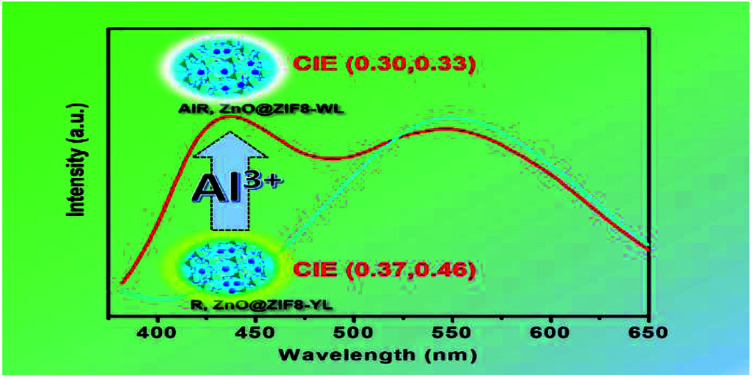

## Introduction

White light emitting diodes (WLEDs) have the advantages of a long life, small size, high efficiency and environmental protection, which are unparalleled to previous generations of solid-state light sources.^1^ There are two main methods for the WLED preparation. The method with the highest efficiency of white light is to combine multiple chips with different light colors; however, it is limited by its high cost and complexity. Another mainstream approach is to match blue or ultraviolet LED chips with their corresponding phosphors. For example, blue LED chips match yellow phosphors and ultraviolet LED chips match red, blue, and green phosphors in proportion.^2^ So far, this is still the mainstream method for preparing white light LEDs because of the high efficiency, simple preparation and low cost. In addition, the phosphors for commercial WLEDs are generally made from inorganic materials that are doped with rare earth ions, but rare earth ions are very expensive.^3,4^ Therefore, the development of rare-earth-free phosphors for WLEDs needs to be carried out; however, it is still a big challenge to design and synthesize more efficient white-light phosphors.

Metal–organic frameworks (MOFs) are assembled from coordinative interactions; it has emerged as a very promising host material depending on its adjustable cavity structure, customizable chemistry and high porosity.^5–7^ The permanent porosity in these structures permits the accommodation of guest molecules within their frameworks, offering a degree of tunability in their emission properties.^8^ Moreover, most fluorescent dyes will encounter aggregation-caused quenching (ACQ) in concentrated solutions or solid crystals. The porous MOFs can facilitate the dispersion of fluorescent dyes effectively and still maintain a good luminescence performance, which make it possible to turn the fluorescent dyes into devices.^9^ Therefore, this simple way of developing MOFs and matching the multicomponent fluorescent dyes to design and fabricate white light emitting materials has been intensively studied in recent years.^10–12^ However, most fluorescent dyes have a smaller Stokes shift, which causes a low fluorescence efficiency and poor anti-interference effect, so it is difficult to balance the various fluorescent dyes to obtain a white light under blue or ultraviolet LED chips.^13^ Thus, an effective method must be developed to obtain the highly efficient and stable white-light emitting MOF-based materials.

With all of this in mind, we considered introducing ZnO quantum dots (QDs) and the fluorescent dye disodium 2-naphthol-3,6-disulfonate (R) into the MOFs. ZnO QDs have a larger Stokes shift and more stable fluorescence properties, which means that there are more options for the wavelength of excitation.^14^ Moreover, the R salt shows an ultra-strong blue emission and it also matches the excitation wavelength range of the ZnO QDs well. This enables a possibility for designing the white-light-emitting material based on those two components. Finally, a fluorescent tunable hybrid material R,ZnO@ZIF-8 is assembled by a “ship around the bottle” method. However, this hybrid material suffered from a low anti-jamming capability and quantum efficiency.

Therefore, we proposed an *in situ* ion regulation strategy to assemble a white-light-emitting material with high stability and efficiency. First, in order to settle the non-tunable problem of fluorescence, which is induced by the over competition and coordination effect between the aluminium ions and zinc ions, a yellow-light-emitting material was fabricated by increasing the ZnO QDs proportion. Then, the competition coordination of the aluminium ions were utilized to exchange the zinc ions with R salts, adjusting the intensity and Stokes shift of the blue fluorescence subtly, leading to an ideal white light with Commission Internationale de l'Eclairage (CIE) coordinates of (0.30, 0.33) and a high Color-Rendering Index (CRI) value of 93%. This synthetic strategy may open a new avenue for designing new white-light-emitting materials.

**Fig. 1:**
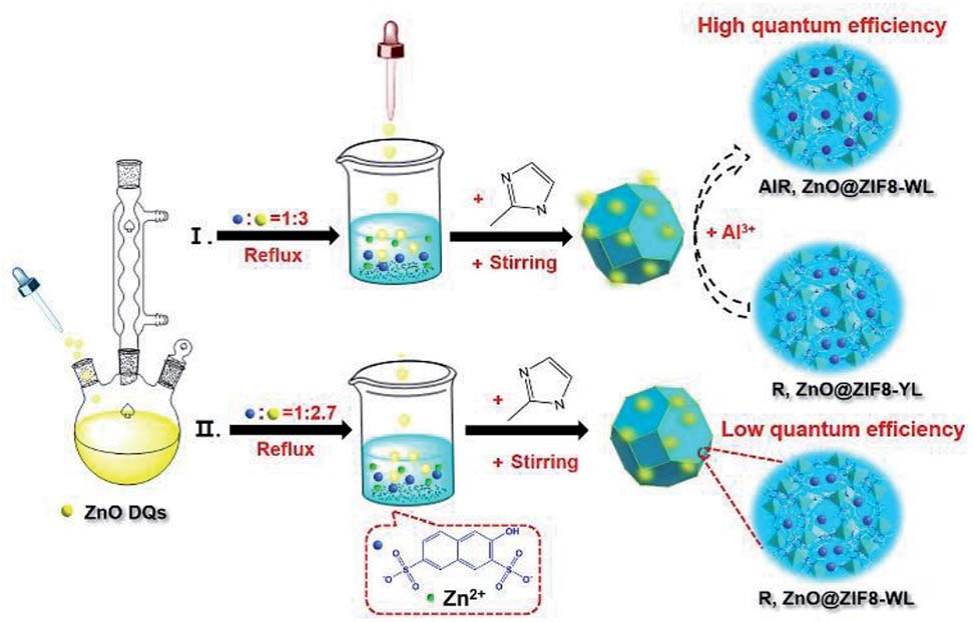
Schematic of the synthesis process for the white light R,ZnO@ZIF8-WL, yellow light R,ZnO@ZIF8-YL, and *in situ* aluminium ions regulation for AlR,ZnO@ZIF8-WL.

ZnO QDs were easily synthesized *via* the addition of a potassium hydroxide alkaline solution into a zinc acetate ethanol solution at 75 °C reflux (see details in the ESI†). The synthesis procedure of R,ZnO@ZIF8-YL (“YL” means yellow light) is shown in Fig. 1. First, the filtered clear R salt was coordinated with the zinc ions, and the volume ratio of R to zinc nitrate was 1 : 1 for enhancing blue light emission at room temperature. A ZnO QDs ethanol solution was then added to the above mixed solution with stirring, keeping the volume ratio of R to ZnO QDs at 1 : 3. Finally, to enclose the luminous guest species into ZIF-8, a 2-methylimidaole (2-MeIM) ethanol solution was injected into the above mixture solution with magnetic stirring for 24 h. The products were dried under vacuum to get the R,ZnO@ZIF8-YL powder. We then added 60 μL of an Al(NO_3_)_3_ ethanol solution (0.1 M) onto the surface of the R,ZnO@ZIF8-YL per 50 mg. This step regulated the ability of the yellow light material to emit white light, and had been verified as the optimal ratio of *in situ* regulation of aluminium ions (see details of proof in Fig. S3–S5, ESI†). This white light material is marked as AlR,ZnO@ZIF8-WL (“WL” means white light). In addition, we adjusted the volume ratio of R to ZnO QDs to obtain 1 : 2.7 in order to get another white light emitting material, R,ZnO@ZIF8-WL.

**Fig. 2:**
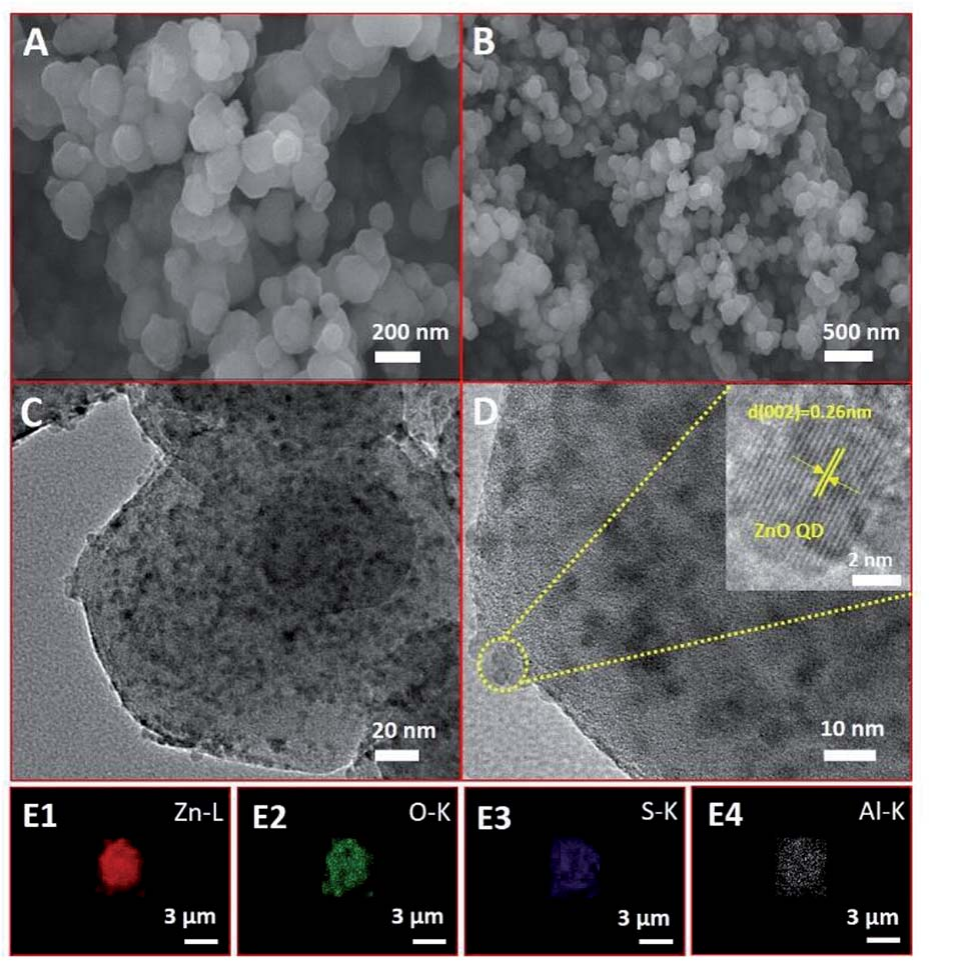
(A) and (B) SEM images of AlR,ZnO@ZIF8-WL. (C) and (D) TEM images of AlR,ZnO@ZIF8-WL (the inset of (D) shows a HRTEM image of a ZnO QD, revealing a lattice spacing of 0.26 nm that arises from the (002) plane of the ZnO QD). (E1)–(E4) Elemental mapping of AlR,ZnO@ZIF8-WL.

From scanning electron microscopy (SEM), it can be seen that the surface of the AlR,ZnO@ZIF8-WL is basically smooth and monodisperse in the size range of 50–100 nm (Fig. 2A and B). Compared with R,ZnO@ZIF8-YL and R,ZnO@ZIF8-WL (Fig. S1A and B, ESI†), the composite materials remained essentially the same. The morphology of the material cannot be affected by the aluminium ion or the concentration of ZnO QDs, which indicated that the material has good stability. Moreover, from high resolution transmission electron microscopy (HRTEM), we also see the distribution of ZnO QDs in the space of ZIF-8 (Fig. 2C and 2D), which were uniformly dispersed at about 5–6 nm in size. The inset of Fig. 2D showed a HRTEM image of a ZnO QD, and it revealed a lattice spacing of 0.26 nm arising from the (002) plane of ZnO QDs. The element mappings (Fig. 2E1–E4) of AlR,ZnO@ZIF8-WL exhibited the homogeneous Zn, O and S elemental distribution over the entire architecture, which demonstrated the good dispersion of the ZnO QDs and fluorescent dye R. Moreover, the distribution of Al element was almost consistent with that of S element, which suggested that there might be a coordination between the aluminium ions and fluorescent dyes.

This is different from the core–shell microsphere structure previously reported for ZnO@ZIF-8. In its special core–shell structure, only one ZnO microsphere of about 100–200 nm was wrapped by ZIF-8, and the thickness of ZIF-8 was about 50–100 nm.^15^ At the same time, it is also different from the ZnO@ZIF-8 obtained by gas-phase deposition; the size of ZnO in the material obtained by this method is subject to the size of the pore channel of ZIF-8, and the content is relatively low.^16^ Therefore, in our unique colloidal semiconductor quantum dot synthesis method,^17^ we could easily introduce a large amount of ZnO QDs into ZIF-8 and keep their monodisperse and luminescence characteristics (Table S2†).

**Fig. 3:**
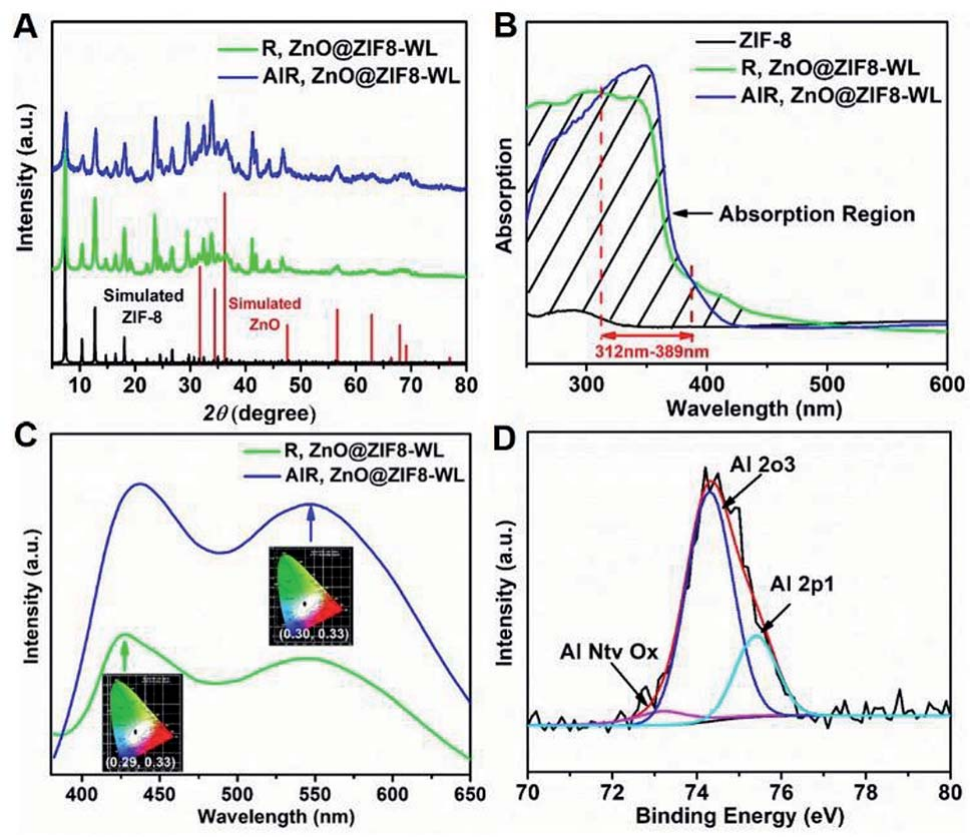
(A) The PXRD of AlR,ZnO@ZIF8-WL and R,ZnO@ZIF8-WL. (B) The solid state UV absorption spectrum of AlR,ZnO@ZIF8-WL, R,ZnO@ZIF8-WL and ZIF-8, respectively. (C) The fluorescence spectrum of R,ZnO@ZIF8-WL and AlR,ZnO@ZIF8-WL at 356 nm. (Inset: the CIE coordinate diagram excited at 356 nm.) (D) XPS spectra of AlR,ZnO@ZIF8-WL.

Moreover, the PXRD patterns of AlR,ZnO@ZIF8-WL and R,ZnO@ZIF8-WL had similar crystalline phases of ZIF-8 (Fig. 3A), indicating that the introduction of guest molecules into ZIF-8 cannot damage the crystalline structure. AlR,ZnO@ZIF8-WL raised the peaks at 56, 63, and 68, which can be indexed to the (110), (103) and (112) crystal planes of the ZnO phase, respectively. These results suggested that the ZnO QDs were conserved. Compared with ZIF-8 nanocrystals, the AlR,ZnO@ZIF8-WL and R,ZnO@ZIF8-WL exhibited a broadband absorption feature in the solid state UV absorption spectrum (Fig. 3B). It can be seen that the ultraviolet absorption of ZIF-8 was changed after the fluorescent dyes and ZnO QDs were loaded. With the addition of the aluminium ions, there was an obvious UV absorption enhancement between 312 nm to 389 nm. At the same time, AlR,ZnO@ZIF8-WL had a stronger fluorescence intensity (Fig. 3C), implying that AlR,ZnO@ZIF8-WL has a higher quantum yield, which is consistent with the following test results (Table S2†).

From the X-ray photoelectron spectroscopy (XPS) peak table (Table S1†), the elemental content of Zn and O in R,ZnO@ZIF8-YL had significantly increased when the volume ratio of ZnO was raised. In addition, the elemental content of Al in R,ZnO@ZIF8-YL was very low, which can be considered negligible. However, a dramatic increase in the elemental content of Al in AlR,ZnO@ZIF8-WL can be observed. The characteristic peak of the Al element appears in the Energy Dispersive X-Ray (EDX) Spectroscopic analysis (Fig. S2, ESI†) of AlR,ZnO@ZIF8-WL, suggesting that the Al element was introduced into ZIF-8. Furthermore, the existence of an Al–O bond was detected by XPS spectra (Fig. 3D) to verify that the aluminium ions were introduced into ZIF-8 and coordinated with the fluorescent dye R.

We excited R,ZnO@ZIF8-WL, R,ZnO@ZIF8-YL and AlR,ZnO@ZIF8-WL between 350–365 nm to obtain a series of excitation spectra, and all composites presented the characteristics of a dual-peak emission (Fig. S6, ESI†). There were two different emission peaks at 428 nm and 550 nm, corresponding to the emission of R–Al(iii)/Zn(ii) and ZnO, respectively. Moreover, the emission intensity of the two peaks had a significant frequency dependence and gradually increased with the redshift of the excitation wavelength at 428 nm (Fig. S6, ESI,† the red arrow is pointing).

**Fig. 4:**
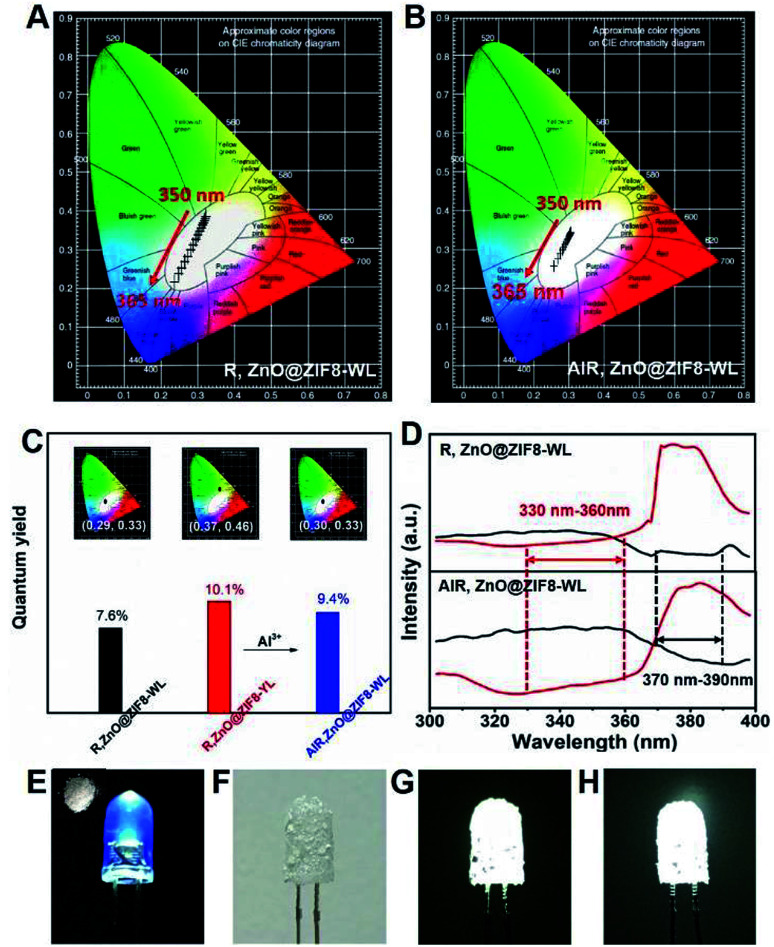
(A) and (B) The CIE coordinates from 350 nm to 365 nm of R,ZnO@ZIF8-WL and AlR,ZnO@ZIF8-WL, respectively. (C) Quantum yields of R,ZnO@ZIF8-WL, R,ZnO@ZIF8-YL and AlR,ZnO@ZIF8-WL. (Inset: individual CIE coordinate diagrams excited at 356 nm of R,ZnO@ZIF8-WL, R,ZnO@ZIF8-YL and AlR,ZnO@ZIF8-WL.) (D) Excitation spectrum of R,ZnO@ZIF8-WL and AlR,ZnO@ZIF8-WL, respectively. (E) Photograph of a 365 nm commercially available ultraviolet LED chip when the LED is turned on. (Inset: dry powder of AlR,ZnO@ZIF8-WL under sunlight.) (F) Photograph of the ultraviolet LED coated with AlR,ZnO@ZIF8-WL when the LED is turn off. (G) The coated LED is turned on. (H) The same WLED placed in the air for one month.

It is worth noting that the CIE coordinates of R,ZnO@ZIF8-WL had a long range moving from 350 nm to 365 nm on a large scale from warm white light to cold white light (Fig. 4A). However, AlR,ZnO@ZIF8-WL moved only in a small range in the white light region (Fig. 4B), which signified that it was less affected by the wavelength. Compared with R,ZnO@ZIF8-WL, the quantum yield of AlR,ZnO@ZIF8-WL had been greatly improved and was as high as 9.4%. In addition, the CEI coordinates (0.30, 0.33) of AlR,ZnO@ZIF8-WL was closest to the white light (Fig. 4C) and had a high CRI value of 93% (Table S3†).^18,19^

In order to explain this phenomenon of increased quantum efficiency, we plotted the excitation spectra of R,ZnO@ZIF8-WL and AlR,ZnO@ZIF8-WL (Fig. 4D), and found that both of them have an intersection to ensure they can be excited at the same time. Moreover, it is remarkable that the AlR,ZnO@ZIF8-WL has a wider excitation range and an intersection in a more favorable excitation position, which ensure that the ZnO QDs and R salt can absorb the excitation energy efficiently with less impact from the excitation wavelength. In addition, to verify that the aluminium ions can enhance the blue luminescence of R, three cuvettes were prepared (Fig. S7, ESI†). The first one is the color of the clarified R ethanol solution under the UV light. To the second cuvette, the same volume of Zn(NO_3_)_2_ ethanol solution (0.05 M) was added. It is clear that the blue luminescence is enhanced in the second one. For the third cuvette, we continued to add the same volume of Al(NO_3_)_3_ ethanol solution (0.05 M), and the blue luminescence was even more enhanced and became dark blue.

In addition, to verify the stability of this *in situ* regulation method, the PXRD measurements were performed. As expected, the PXRD shown in Fig. S8† indicate that the addition of aluminium ions did not destroy the crystalline structure of ZIF-8. As a result, the method of *in situ* regulation has been proven to be feasible, and provided a new way to regulate the fluorescence emission for other solid-state fluorescent materials. In order to test the material for practical white-light-emission applications, the WLED assemblies were fabricated by simply coating the AlR,ZnO@ZIF8-WL powders onto a commercially available ultraviolet LED chip (5 mm, 365 nm).^10,18,20^ Under a voltage of 3.8 V, as illustrated in Fig. 4E–G, the assembled WLED displayed a bright white light. Furthermore, the AlR,ZnO@ZIF8-WL powders can maintain high stability; when the assembled LED chip was placed in the air for a month, it could still maintain a bright white light (Fig. 4H). For comparison, the fluorescent intensity of AlR,ZnO@ZIF8-WL shows a slighter change under different excitation wavelength, which indicates a higher stability than R,ZnO@ZIF8-WL (Fig. S6 and S9 ESI†). Therefore, the utilization of R,ZnO@ZIF8 as a solid-state stable and environment-friendly phosphor has potential applications in WLEDs.

In summary, we have demonstrated a simple “ship around the bottle” method for assembling a novel, stable and green solid-state white-light-emitting material. Moreover, an *in situ* ion regulation strategy was utilized to promote the fluorescence stability and efficiency by the competition coordination effect (CIE coordinates = (0.30, 0.33), CRI value = 93%). Finally, this hybrid material AlR,ZnO@ZIF8-WL could be assembled as a WLED device and kept stable for over one month in ambient conditions. We believe that this synthetic strategy can provide more avenues for designing new white-light-emitting materials.

## Conflicts of interest

There are no conflicts to declare.

## Supplementary Material

RA-009-C9RA01763A-s001
