# Supplementary material for: In situ aluminium ions regulation for quantum efficiency and light stability promotion in white light emitting material
Source: RSC Adv. 2019 May 17;9(27):15265–8. doi: 10.1039/c9ra01763a (PMC9064237; doi:10.1039/c9ra01763a)
Supplement: RA-009-C9RA01763A-s001 [file RA-009-C9RA01763A-s001.pdf]

Electronic Supplementary Material (ESI) for RSC Advances.
This journal is © The Royal Society of Chemistry 2019

Supporting Information

In Situ Aluminium Ions Regulation for Quantum Efficiency and Light Stability Promotion in White Light Emitting Material

*Qian Liu [a,+], Qi-Long Wu [a,+], Man-Xiu Nie [a], Da-Shuai Zhang [b], Jiong-Peng Zhao *[a] and Fu-Chen Liu *[a]*

[a] Q. Liu, Dr. Q.-L. Wu, Dr. M.-X. Nie, Prof. J.-P. Zhao, Prof. F.-C. Liu
School of Chemistry and Chemical Engineering,
Tianjin University of Technology
Tianjin 300384, China.
E-mail: zhaojp@tjut.edu.cn; fcliu@tjut.edu.cn
[b] College of Chemistry and Chemical Engineering, Dezhou University, Dezhou, 253023, P. R. China.
[+]These authors contributed equally to this work.

Supplementary Section 1. Materials and instrumentation

Supplementary Section 2. Supplementary Figs

Supplementary Section 3. Supplementary Tables

Supplementary Section 1. Materials and instrumentation

S1.1 Materials and characterization

All the reagents and solvents used for the synthesis were commercially available and used without further purification. $Zn(CH_3COO)_2·2H_2O$, KOH, $Zn(NO_3)_2·6H_2O$, disodium 2-naphthol-3,6-disulfonate (referred to as “R”), 2-methylimidazole (2-MeIM) and $Al(NO_3)_3$ were purchased from Tianjin Heowns Biochemical Technology Co., Ltd. Ethanol was acquired from Tianjin Chemical Reagent Co., Ltd.

The Powder X-ray diffraction (PXRD) acquired using a Rigaku Ultima IV. Scanning electron microscope (SEM, ZEISS MERLIN Compact) and transmission electron microscope (TEM, Talos F200X) were used to observe the morphology. X-ray photoelectron spectroscopic (XPS) spectra were collected on a Thermo Scientific Escalab 250 Xi XPS spectrometer. UV absorption spectrum was measured on a Hitachi 3900 UV-vis spectrophotometer at room temperature. A Hitachi F4500 fluorescence spectrometer was used for the photoluminescence measurement. NETZSCH TG 209 F3 was used to conduct thermogravimetric analysis. CIE calculator-version 3 software was utilized to calculate CIE chromaticity coordinates, CRI, and CCT.

S1.2 Synthesis of ZnO quantum dots (ZnO QDs) -EtOH solution: $Zn(CH_3COO)_2·2H_2O$ (0.384 g) were dissolved in 35 mL of ethanol and then magnetic stirred about 30 min at 75℃ until the zinc solution turn to clarifying, and then 1.5 mL of KOH ethanol solution (2 mol L^{-1}) was added into the above solution under magnetic stirred at 75℃ for 6 min and a clear solution was obtained.

S1.3 Synthesis of R,ZnO@ZIF8-WL: R (50 mg) was dissolved in 40 mL of ethanol, and filtered through a 0.22 μm filter to get 10 mL of the filtrate. And $Zn(NO_3)_2·6H_2O$ (0.147 g) was dissolved in 10 mL ethanol, 2-MeIM (0.168 g) was dissolved in 5 mL ethanol, respectively. Then 10 mL $Zn(NO_3)_2·6H_2O$ ethanol solution was added into above 10 mL R ethanol solution firstly with magnetic stirred for 10 min at the room temperature, and then 27 mL ZnO QDs was injected into above solution. Finally, 5 mL 2-MeIM ethanol solution was poured into the above solution

with the mixture solution was magnetic stirred for 24 h. The product was collected by centrifuge (8000 rpm, 2 min), then the product was washed three times by EtOH and dried in vacuum at 50 ºC for 6 h.

S1.4 Synthesis of R,ZnO@ZIF8-YL: The synthesis steps are same with **R,ZnO@ZIF8-WL**, except that the volume of ZnO QDs is 30 mL. The obtained powder was washed three times by EtOH and dried in vacuum at 50 ºC for 6 h.

S1.5 Synthesis of AlR,ZnO@ZIF8-WL: 60 μL $Al(NO)_3$ ethanol solution (0.1 mol L^{-1}) was dropped on the surface of **R,ZnO@ZIF8-YL** powder per 50 mg. Finally, the obtained powder was dried in vacuum at 50 ºC for 6 h.

S1.6 Synthesis of ZnO@ZIF-8: $Zn(NO_3)_2{\cdot}6H_2O$ (0.147 g) was dissolved in 20 mL of ethanol, and 2-MeIM (0.168 g) was dissolved in 5 mL ethanol. Then 20 mL ethanol solution of zinc nitrate was dropped into 27 mL ZnO QDs under magnetic stirred. Finally, 5 mL 2-MeIM ethanol solution was poured into the above solution with the mixture solution was magnetic stirred for 24 h.

S1.7 Synthesis of R@ZIF-8: R (50 mg) was dissolved in 40 mL of ethanol, and filtered through a 0.22 μm filter to get 10 mL of the filtrate. And Zn(NO3)2•6H2O (0.294 g) was dissolved in 37 mL ethanol, 2-MeIM (0.168 g) was dissolved in 5 mL ethanol, respectively. Then 37 mL ethanol solution of zinc nitrate was dropped into 10 mL of R filtrate under magnetic stirred. And 5 mL 2-MeIM ethanol solution was poured into the above solution. Finally, 1 mL of KOH ethanol solution (2 mol L^{-1}) was added into the above solution under magnetic stirred for 24 h.

S1.8 Colour temperature (CCT) calculation

The CCT values were calculated using the following equation (1):

$$T = 437n^3 + 3601n^2 + 6831n + 5517 \qquad (1)$$

Where $n = \frac{x - 0.3320}{0.1858 - y}$, and (x, y) is the value of CIE.

Supplementary Section 2. Supplementary Figs

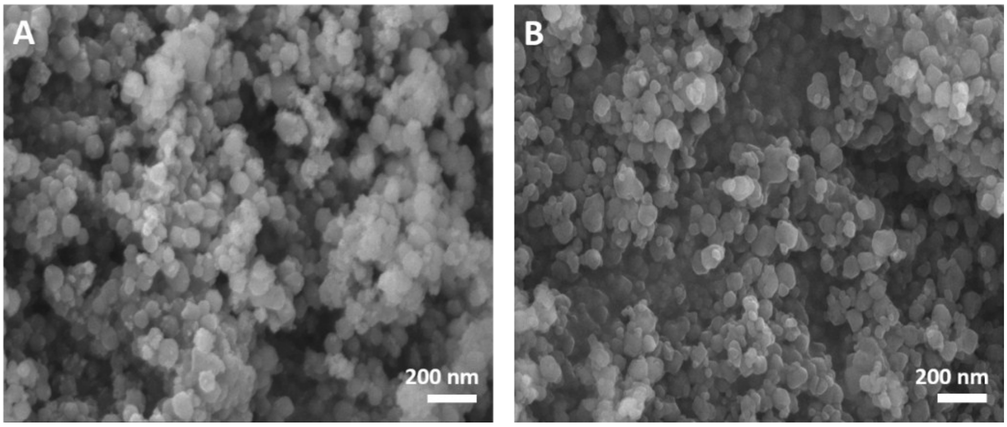

Fig. S1 SEM of (A) R,ZnO@ZIF8-WL and (B) R,ZnO@ZIF8-YL.

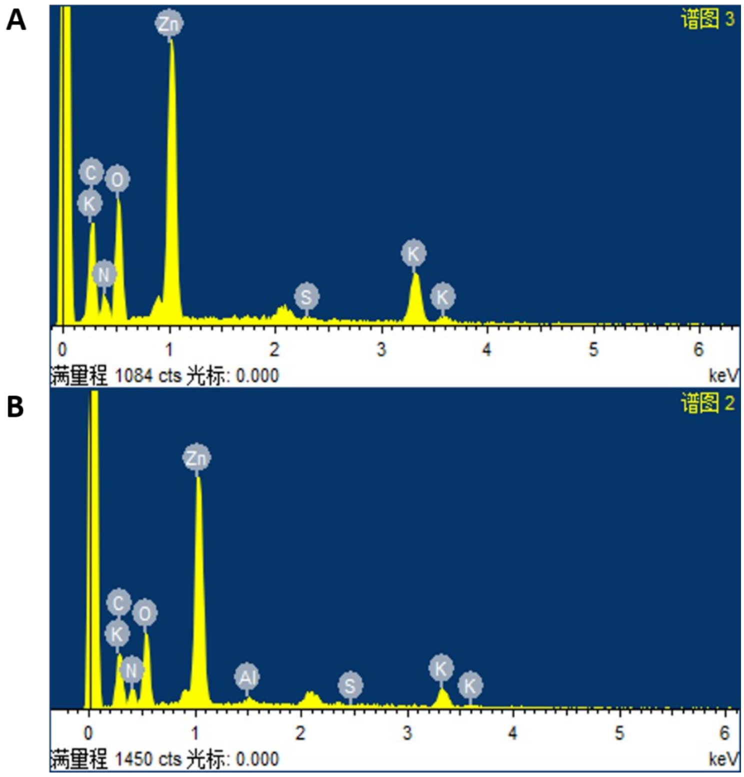

Fig. S2 The EDX spectrum of (A) R,ZnO@ZIF8-WL and (B) AlR,ZnO@ZIF8-WL.

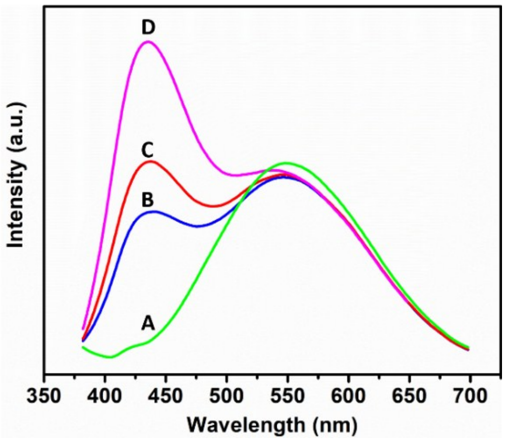

Fig. S3 The fluorescence spectrum under 356 nm excitation of (A) R,ZnO@ZIF8-YL. (B) Dropped 30μL $Al(NO_3)_3$ ethanol solution (0.1 M) on the surface of R,ZnO@ZIF8-YL per 50 mg, marked as AlR,ZnO@ZIF8-30. (C) Dropped 60 μL $Al(NO_3)_3$ ethanol solution (0.1 M) on the surface of R,ZnO@ZIF8-YL per 50 mg, marked as AlR,ZnO@ZIF8-60. (D) Dropped 90 μL $Al(NO_3)_3$ ethanol solution (0.1 M) on the surface of R,ZnO@ZIF8-YL per 50 mg, marked as AlR,ZnO@ZIF8-90.

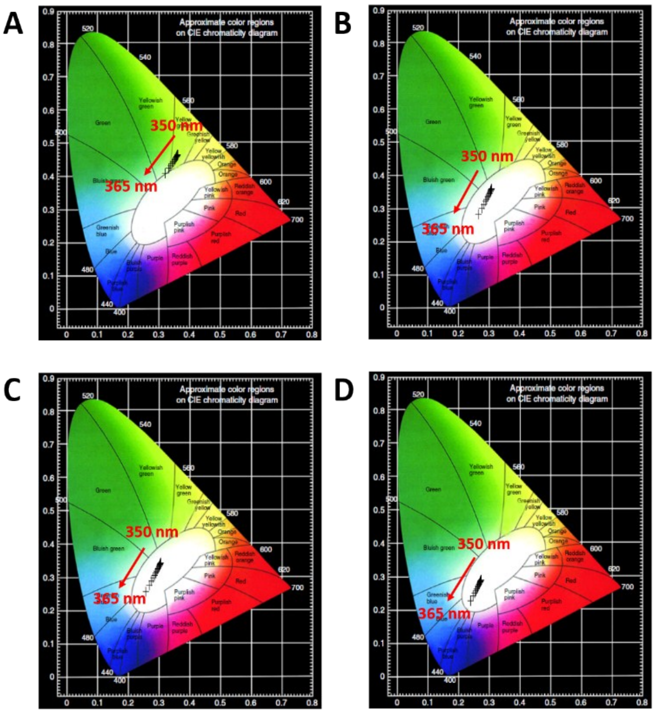

Fig. S4 CIE coordinates from 350 nm to 365 nm of (A) R,ZnO@ZIF8-YL, (B) AlR,ZnO@ZIF8-30, (C) AlR,ZnO@ZIF8-60 and (D) AlR,ZnO@ZIF8-90, respectively.

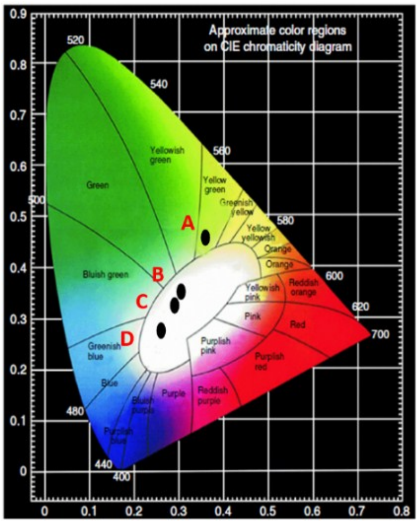

Fig. S5 CIE coordinates at 356 nm of (A) R,ZnO@ZIF8-YL, (B) AlR,ZnO@ZIF8-30, (C) AlR,ZnO@ZIF8-60 and (D) AlR,ZnO@ZIF8-90, respectively.

The optimal ratio of vitro regulation of aluminium ions had be tested. When we increased drop $Al(NO_3)_3$ ethanol solution (0.1 M) from 30 μL to 90 μL on the surface of the R,ZnO@ZIF8-YL per 50 mg, the relative peak intensity continuously increased at 428 nm of fluorescence spectrum (Fig. S3), demonstrated that the aluminium ions are easier to coordinate with R than zinc ions and can enhance its blue light emission. We can clearly observe the change of CIE coordinates from 350 nm to 365 nm with the volume of the droplet changes (Fig. S4), the R,ZnO@ZIF8-YL fell in the yellow-green light region, and with the increase of the droplet content gradually move towards blue light region, passing through the white light area. From the CIE chromaticity diagram (Fig. S5), we can see the different drop conditions with the CIE coordinates can be shifted from (0.37, 0.46) to (0.27, 0.28) under 356 nm excitation. And we can draw a conclusion that the best condition of white light emission was to drop 60 μL of aluminium nitrate ethanol solution on the surface of R,ZnO@ZIF8-YL per 50 mg.

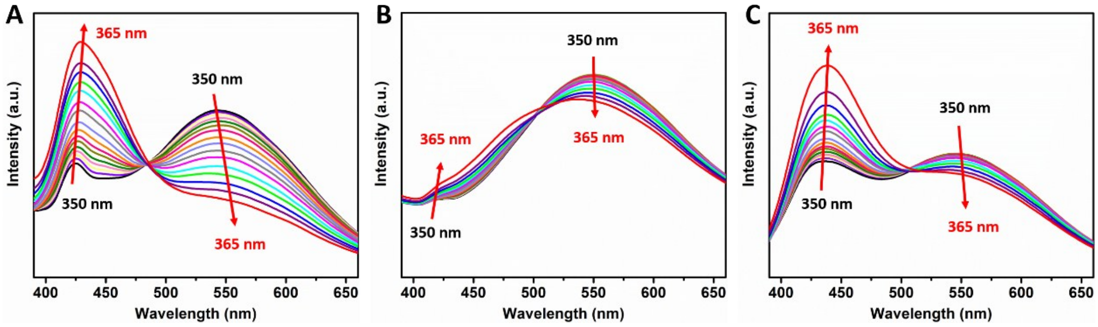

Fig. S6 The fluorescence spectrum of (A) R,ZnO@ZIF8-WL, (B) R,ZnO@ZIF8-YL and (C) AlR,ZnO@ZIF8-WL at an excitation wavelength from 350 nm to 365 nm (1 nm per date point), respectively.

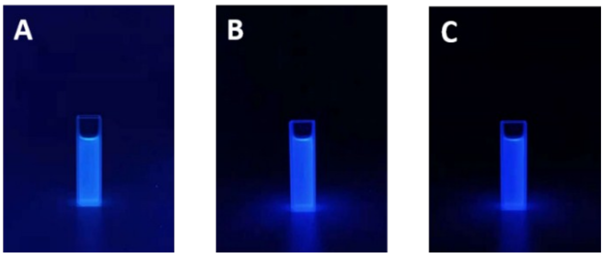

Fig. S7 The picture of continuous solution color under the excitation of UV light, and for the sake of contrast, we kept the total volume of the solution in the cuvettes basically the same, all the solutions were mixed and then added back to the cuvettes. (A) Clear ethanol dispersion solution of R after 0.22 μm filter. (B) An ethanol solution of $Zn(NO_3)_2$ (0.05 M) in the same volume was added to A. (C) Continue to add the same volume of $Al(NO_3)_3$ (0.05 M) ethanol solution to B.

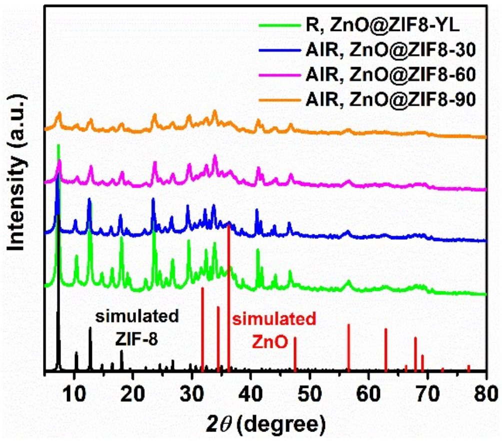

Fig. S8 PXRD patterns of R,ZnO@ZIF8-YL, AlR,ZnO@ZIF8-30, AlR,ZnO@ZIF8-60 and AlR,ZnO@ZIF8-90, respectively.

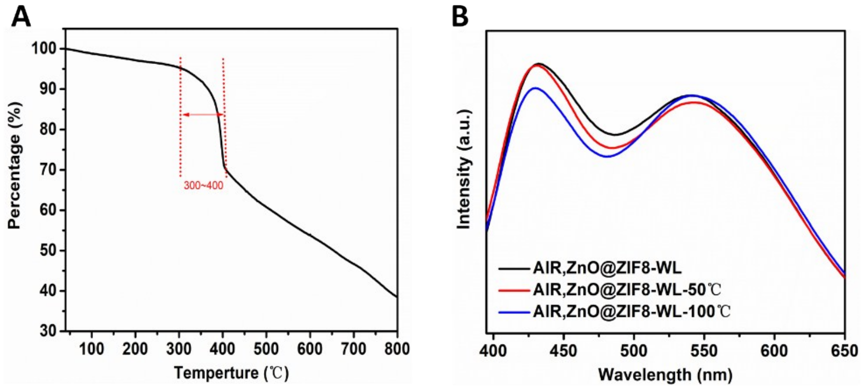

Fig. S9 Thermogravimetric analysis (A) and the fluorescence spectrum (B) of AlR,ZnO@ZIF8-WL.

In order to study the thermal-stability of the AlR,ZnO@ZIF8-WL material, we used a thermogravimetric analyzer to conduct a thermogravimetric analysis. The temperature range of the instrument was set at 40 ~ 800°C, the heating rate was 10°C/min, the gas atmosphere was nitrogen, and the flow rate was 20 mL/min. we can see that the material causes only 6% mass loss at 300°C, and a sharp loss of mass was caused when the temperature rose to 400°C. Therefore, we heated the sample to a certain temperature in a vacuum drying oven, and restored it to room temperature to conduct fluorescence spectrum tests. We can see the AlR,ZnO@ZIF8-WL under 50°C/100°C treatment, the fluorescence spectrum shape of AlR,ZnO@ZIF8-WL basic remain unchanged. So we can think the material have good thermal-stability.

Supplementary Section 3. Supplementary Tables

Table S1. Element distribution of R,ZnO@ZIF8-WL, R,ZnO@ZIF8-YL and AlR,ZnO@ZIF8-WL (based on XPS).

Materials	Zn (Atomic %)	O (Atomic %)	S (Atomic %)	Al (Atomic %)
R,ZnO@ZIF8-WL	3.35	6.27	0.45	0.6
R,ZnO@ZIF8-YL	8.05	12.86	0.73	0.91
AlR,ZnO@ZIF8-WL	5.17	39.11	0.57	5.5

Table S2 The Quantum yield of R, ZnO QDs, R@ZIF8, ZnO@ZIF8, R,ZnO@ZIF8-WL and AlR,ZnO@ZIF8-WL.

Materials	Quantum yield (%)
R	1.9
Separated ZnO QDs in solid state	2.0
R@ZIF8	12.3
ZnO@ZIF8	13.7
R,ZnO@ZIF8-WL	7.6
AlR,ZnO@ZIF8-WL	9.4

Table S3 The CEI, CCT and CRI values of R,ZnO@ZIF8-WL and AlR,ZnO@ZIF8-WL.

Materials	CEI	CCT/K	CRI
R,ZnO@ZIF8-WL	(0.29, 0.33)	7570	91

AlR,ZnO@ZIF8-WL	(0.30, 0.33)	7237	93
